# Supplementary material for: “I can be a source of motivation”: Perspectives from stakeholders of the I’mPossible fellowship, a peer-led differentiated service delivery model for adolescents with perinatally acquired HIV in India
Source: PLOS Glob Public Health. 2025 Sep 4;5(9):e0004453. doi: 10.1371/journal.pgph.0004453 (PMC12410805; doi:10.1371/journal.pgph.0004453)
Supplement: S1 Text — (DOCX) [file pgph.0004453.s001.docx]

Qualitative Codebook

Individual In-depth Interviews

| Root Code | Parent Code | Parent Code Definition | Child Code | Child Code Definition |
| --- | --- | --- | --- | --- |
| Facilitators to the Fellowship | Individual Factors | Personal attributes or internal motivations that enable a fellow to successfully participate in the fellowship. | Personal motivation and goals | The fellow’s intrinsic drive, aspirations, or sense of purpose related to joining or succeeding in the program. |
|  |  |  | Self-efficacy and resourcefulness | Confidence in one’s ability to manage challenges, solve problems, and take initiative independently. |
|  |  |  | Positive mental health and emotional well-being | Feelings of stability, resilience, and optimism that support participation. |
|  | Interpersonal Factors | Influence of relationships and social connections in supporting engagement with the fellowship. | Positive peer influence and social networks | Encouragement, motivation, or shared experiences from peers that support involvement. |
|  |  |  | Mentorship and guidance from role models | Support and inspiration received from mentors, staff, or senior fellows. |
|  | Community Factors | Aspects of the local or institutional environment that foster a sense of support and opportunity. | Positive reputation and perceived benefits | Community recognition of the fellowship’s value and benefits for participants. |
|  |  |  | Supportive and inclusive community environment | Presence of an enabling space where fellows feel welcomed, valued, and understood. |
|  | Societal Factors | Broader societal changes or systems that reduce barriers to participation. | Decreased HIV/AIDS stigma and discrimination | Reduction in public prejudice or judgment that allows fellows to engage openly. |
|  |  |  | Access to healthcare and social services | Availability of external support systems that meet health and social needs. |
| Barriers to the Fellowship | Individual Factors | Personal challenges or constraints that hinder participation or motivation. | Transportation and logistical challenges | Difficulty in reaching program sites or managing schedules due to practical issues. |
|  |  |  | Health concerns | Physical health issues that affect attendance or performance. |
|  |  |  | Fear of disclosure and stigma | Anxiety about others discovering one's HIV status or facing judgment. |
|  |  |  | Mental health or emotional challenges | Psychological struggles such as anxiety, depression, or trauma. |
|  |  |  | Not feeling confident in abilities | Doubts about one’s capacity to succeed or contribute meaningfully. |
|  |  |  | Time constraints | Limited availability due to other responsibilities like school, work, or caregiving. |
|  | Interpersonal Factors | Negative or unsupportive dynamics with family, peers, or others. | Lack of family support or understanding | Absence of encouragement or resistance from family members. |
|  |  |  | Negative peer influence or social pressure | Discouragement or harmful behaviors from peers that deter participation. |
|  |  |  | Conflicts with family or personal commitments | Incompatibility between program demands and family or personal expectations. |
|  | Community Factors | Barriers emerging from institutional, organizational, or environmental structures. | Problems with institutional placement | Poor fit, lack of clarity, or logistical issues at program sites. |
|  |  |  | Lack of sensitivity to diverse needs | Insufficient accommodation for fellows’ identities, backgrounds, or experiences. |
|  |  |  | Bureaucratic hurdles and administrative challenges | Unclear processes, or systemic inefficiencies within the program. |
|  | Societal Factors | Structural and cultural conditions that perpetuate exclusion or inequality. | Persistent HIV/AIDS stigma and discrimination | Continued societal prejudice that creates unsafe or isolating conditions. |
|  |  |  | Economic inequalities and limited resources | Poverty, lack of materials, or systemic disinvestment in communities. |
|  |  |  | Discriminatory healthcare policies or practices | Unequal treatment or access in health systems due to HIV status, gender, caste, etc. |
| Perspectives on the Fellowship | Impact on individual development | Changes in self-perception, abilities, and learning as a result of participating. | Increased self-esteem and confidence | Greater belief in one’s worth and abilities. |
|  |  |  | Improved academic achievement and educational aspirations | Enhanced performance or future plans related to education. |
|  |  |  | Development of leadership skills and communication abilities | Growth in capacity to guide others and express oneself clearly. |
|  |  |  | Increased HIV/AIDS knowledge and health management skills | Improved understanding of HIV and how to care for one’s health. |
|  |  |  | Enhanced social and emotional well-being | Strengthened coping mechanisms, emotional regulation, and social support. |
|  | Impact on relationships | Shifts in how fellows relate to family, peers, and community. | Strengthened family relationships | Improved bonds or communication with family members. |
|  |  |  | Development of supportive peer networks | Formation of encouraging and meaningful peer connections. |
|  |  |  | Enhanced communication and interpersonal skills | Increased ability to listen, empathize, and navigate social settings. |
|  | Impact on future goals | Influence of the fellowship on long-term aspirations and direction. | Clarified educational and career aspirations | Better understanding of desired career or study path. |
|  |  |  | Development of advocacy skills and commitment to activism | Growth in ability and desire to speak up for self and others. |
|  |  |  | Improved personal and professional development plans | More concrete planning for one’s future. |
|  |  |  | Increased sense of hope and self-efficacy | A stronger belief that one’s goals are achievable. |

Focus Group Discussions

| **Area of exploration** | **ROOT** | **PARENT** | **CHILD** | **GRANDCHILD** | **Dictionary** |
| --- | --- | --- | --- | --- | --- |
| 1. Fellowship program awareness | Through people involved in the fellowship program | Fellows |  |  | Refers to if peers heard of the I'mPossible Fellowship Program through members involved in the program, i.e., fellows in the programs or supervisors |
|  |  | Seniors/Supervisors |  |  |  |
|  | Through outside means | Other programs |  |  | Refers to if peers heard of the I'mPossible Fellowship Program through other programs that they partook in |
| 2. Interactions with fellows | Peers' specific interactions with fellows | Academic assistance |  |  | Refers to if fellows helped peers to academic matters, i.e. studying for exams, assignments, etc. |
|  |  | Medical/HIV-related support | Tangible support | Medication adherence | Refers to if fellows helped peers with matters related to medical issues, including any HIV-related concerns and provided tangible support in the form of reminding peers to take their medications, assisting peer at the hospital, etc. |
|  |  |  |  | Increasing treatment knowledge |  |
|  |  |  |  | Accompanying peers to ART centers/ caring for them in ill-health |  |
|  |  |  |  | Addressing other health-related concerns |  |
|  |  |  | Emotional support |  | Refers to if fellows provided emotional support to the peers to address their HIV-related problems |
|  |  | Resource to talk to | Talk to about personal matters | Yes | Refers to if fellows were generally described as a resource that peers felt that they would take to about any personal matters |
|  |  |  |  | No |  |
|  | Peers' feelings about interactions with fellows | Supported/Understood | Yes | Because fellows share HIV positive status | Refers to if peers felt supported and understood after their interactions/conversations with the fellows and if this feeling was associated because the fellows were HIV+ as well |
|  |  |  | No |  |  |
|  |  | Increased confidence | Yes |  | Refers to if peers felt increased confidence after their interactions/conversations with the fellows |
|  |  |  | No |  |  |
|  |  | Hesitant to share/talk to them | Yes |  | Refers to if peers felt hesitant to share information or talk to the fellows |
|  |  |  | No |  |  |
| 3. Perspectives on the fellowship program | Community problems | Lack of parental care/support | Yes |  | Refers to if peers mentioned challenges such as lack of parental care or support |
|  |  |  | No |  |  |
|  |  | Lack of employment | Yes |  | Refers to if peers mentioned challenges such as lack of employment or findings jobs |
|  |  |  | No |  |  |
|  |  | Lack of educational support | Yes |  | Refers to if peers mentioned challenges such as lack of support in the matter of education, continuation of studies, etc. |
|  |  |  | No |  |  |
|  |  | Lack of medical support | Yes |  | Refers to if peers mentioned challenges such as lack of medical support either in the conversation of HIV-related care or in general |
|  |  |  | No |  |  |
|  |  | Stigma because of HIV status | Yes |  | Refers to if peers mentioned societal stigma they have faced/witnessed because of their/one's HIV status |
|  |  |  | No |  |  |
|  | Fellowship program additions to address community problems | Provide support for housing |  |  | Refers to if peers mentioned support for housing as a means to mitigate the problems the community faces |
|  |  | Provide opportunities for education |  |  | Refers to if peers mentioned educational support/provision of educational opportunities as a means to mitigate the problems the community faces |
|  | Experience in the fellowship | Positive experience | Yes | Friendships | Refers to if peers expressed a general positive sentiment when discussing the fellowship experience and if the positive sentiment was attributed to the friendships/connections they have made |
|  |  |  | No |  |  |
|  | Impact of fellowship | Increased confidence |  |  | Refers to if peers said they felt increased confidence, boosted self-esteem, etc. because of the fellowship |
|  |  | Gained hard/softs skills |  |  | Refers to if peers said they felt they gained skills (tangible, communication, interpersonal, etc.) because of the fellowship |
|  |  | Increased opportunities | Educational opportunities |  | Refers to if peers said they have received increased educational and/or employment opportunities because of the fellowship |
|  |  |  | Employment opportunities |  |  |
| 4. Future involvement | Interested in becoming a fellow | Yes |  |  | Refers to if peers affirmed they would be interested in becoming a fellow in the future |
|  |  | Maybe | General nervousness |  | Refers to if peers expressed hesitation at the idea of becoming a fellow in the future |
|  |  | No | Fear regarding their health |  | Refers to if peers said they would not be interested in becoming a fellow in the future because of certain fears |
|  |  |  | Fear regarding disclosing their HIV status to others |  |  |
| 5. Mental Health | Source of stress/worry | The future | Uncertain about life after leaving Snehagram | Fear of losing support | Refers to if peers cited their specific worry about the future to be the uncertainty of their life after leaving the program |
|  |  |  | Uncertain about making money |  | Refers to if peers cited their specific worry about the future to be the uncertainty of how they would make money |
|  |  |  | Uncertain about finding employment |  | Refers to if peers cited their specific worry about the future to be the uncertainty of how they would find a job |
|  |  |  | Worried about lack of familial support/care |  | Refers to if peers cited their specific worry about the future to be the uncertainty about familial support |
|  |  | Education | Yes |  | Refers to if peers mentioned their stress is due to their studies, exams, etc. |
|  |  |  | No |  |  |
|  |  | Positive HIV status | Concerned about health challenges/HIV management | Yes | Refers to if peers mentioned their stress is due their positive HIV status and associated health concerns |
|  |  |  |  | No |  |
|  |  |  | Hesitant to discuss HIV-related problems | Yes | Refers to if peers mentioned their stress is due their positive HIV status and feeling hesitant to discuss HIV-related problems for fear of disclosure, etc. |
|  |  |  |  | No |  |
|  |  |  | Feel stigmatized in society | Yes | Refers to if peers mentioned their stress is due their positive HIV status and feeling stigmatized because of this reality |
|  |  |  |  | No |  |
|  |  | Lack of money | Yes |  | Refers to if peers mentioned their stress is due to lack of money, financial troubles, etc. |
|  |  |  | No |  |  |
|  |  | Lack of familial support | Yes | Due to HIV status | Refers to if peers mentioned their stress is due to lack of familial support due to their HIV status |
|  |  |  | No |  |  |
|  | Coping with stress/worry | Inner-resilience |  |  | Refers to if peers exhibit high inner-resilience, self-efficacy, self-esteem in response to these challenges |
|  |  | Self-care/relaxation |  |  | Refers to if peers practice self-care and relaxation methods in response to these challenges, such as listening to music or sleeping |
|  |  | Social support | Talking to friends/peers | Peers with HIV | Refers to if peers seek out social support in response to these challenges, such as friends (those who are HIV positive like them because it is more comfortable) or family members. |
|  |  |  | Talking to family |  |  |
|  |  | Medical support |  |  | Refers to if peers seek out medical support from doctors in response to their challenges |
|  |  | Distraction |  |  | Refers to if peers aim to distract themselves from their challenges through playing games, etc. |
|  | Talking about mental health with others | Barriers | Lack of trust |  | Refers to if peers mentioned that they felt unable to speak to others about mental health issues due to a lack of trust |
|  |  |  | Fear of judgement |  | Refers to if peers mentioned that they felt unable to speak to others about mental health issues due to a fear of judgement |
|  |  |  | HIV-negative status |  | Refers to if peers mentioned that they felt unable to speak to others about mental health issues because they did not share a positive HIV status and felt less comfortable |
|  |  | Enabling factors | Established support |  | Refers to if peers mentioned that they felt able to speak to others about mental health issues because there was a sense of mutual support |
|  |  |  | Shared HIV-positive status |  | Refers to if peers mentioned that they felt able to speak to others about mental health issues because they shared a positive HIV status and felt more comfortable |
|  | Stigma | Personalized stigma |  |  | Refers to if peers mentioned the perceived consequences of other people knowing about their HIV status |
|  |  | Disclosure concerns |  |  | Refers to if peers mentioned their concerns or worries about disclosing their HIV status |
|  |  | Negative self-image |  |  | Refers to if peers shared negative feelings towards themselves due to HIV |
|  |  | Concern with public attitudes |  |  | Refers to if peers spoke about people’s attitudes towards people with HIV |
|  |  | Enacted stigma |  |  | Refers to if peers mentioned external stigma/discrimination/experience of unfair treatment by others due to their HIV status |
|  | Experiences in school | Disclosed HIV status to peers | Yes |  | Refers to if peers mentioned that they had disclosed their HIV status to their peers at school and if a fear of losing friends deterred them from doing so |
|  |  |  | No | Fear of losing friends |  |
|  |  | Discrimination because of HIV status | Yes |  | Refers to if peers spoke about how they were mistreated at school due to their HIV status |
|  |  |  | No |  |  |
|  |  | Comfort in sharing mental health/health-related problems | Yes |  | Refers to if peers said they felt comfortable talking about personal matters/mental health-related matters with peers at school and if fear of HIV status disclosure deterred them from these conversations |
|  |  |  | No | Fear regarding disclosing their HIV status to others |  |
|  | Taking medications | Stressful experience | Yes | Fear of others watching/taking medications in public | Refers to if peers described taking HIV medications as a stressful experience and if it was due to fear of other people knowing/watching them |
|  |  |  | No |  |  |
|  |  | Adherence to medication | Yes |  | Refers to if peers were adherent to their HIV medications and if not, possible deterrents for why this was the case |
|  |  |  | No | Forget to take medication |  |
|  |  |  |  | Difficult to swallow medication |  |
|  | Important relationships in peers' lives | Family |  |  | Refers to if peers cited family or friends, specifically at Snehagram and outside of Snehagram, as their most important relationships in their lives |
|  |  | Friends | At Snehagram |  |  |
|  |  |  | Outside of Snehagram |  |  |
| 6. Substance Use | Witnessed substance use | Family | Yes | Cigarette/tobacco | Refers to if peers witnessed their family members using substances and if yes, what the specific substances were |
|  |  |  |  | Alcohol |  |
|  |  |  |  | Gutka |  |
|  |  |  |  | Other |  |
|  |  |  | No |  |  |
|  |  | People in the program | Yes | Cigarette/tobacco | Refers to if peers witnessed people in the program using substances and if yes, what the specific substances were |
|  |  |  |  | Alcohol |  |
|  |  |  |  | Gutka |  |
|  |  |  |  | Other |  |
|  |  |  | No |  |  |
|  | Frequency of seen substance use among people in program | Low frequency |  |  | Refers to if peers witnessed people in the program using substances at a high frequency, i.e., daily or weekly |
|  |  | High frequency |  |  | Refers to if peers witnessed people in the program using substances at a low frequency, i.e., monthly |
|  | Perceived reason for seen substance use | Enjoyment |  |  | Refers to if peers perceived the reason that people use substances (in the program and in general) to be out of enjoyment |
|  |  | Coping mechanism (due to stress) |  |  | Refers to if peers perceived the reason that people use substances (in the program and in general) to be out of stress and worry–acting as a coping mechanism |
|  |  | Peer pressure/influence |  |  | Refers to if peers perceived the reason that people use substances (in the program and in general) to be from seeing their friends engaging in similar behaving and feeling influence/pressured by them |
|  | Support that should be given to those in the program who use substances | Emotional support |  |  | Refers to if peers mentioned that a means to mitigate substance use is through providing emotional support by talking to them |
