# Supplementary material for: “I can be a source of motivation”: Perspectives from stakeholders of the I’mPossible fellowship, a peer-led differentiated service delivery model for adolescents with perinatally acquired HIV in India
Source: PLOS Glob Public Health. 2025 Sep 4;5(9):e0004453. doi: 10.1371/journal.pgph.0004453 (PMC12410805; doi:10.1371/journal.pgph.0004453)
Supplement: Attachment — Submitted filename: PLOS GPH Review.docx [file pgph.0004453.s003.docx]

This paper stands out for its robust design: a multilayered approach to studying how ALHIV (adolescents living with HIV) navigate and benefit from a peer-led mentorship and support program, implemented within a differentiated service delivery model (DSD) designed specifically to meet the needs of adolescents living with HIV with regard to HIV care. Other innovative elements of this paper are the focus on adolescents with perinatally-acquired HIV infection, as these studies are somewhat rare, and the use of a ‘fellowship’ model for the APHIV youth who deliver the intervention (the I’m Possible fellowship).

The qualitative research design includes in-depth interviews with n=8 adolescent intervention deliverers (fellows), their supervisors and mentors (n=7), and 3 focus group discussions with beneficiaries (the adolescents living with HIV who participated in the program). The data were analyzed with a standard thematic analysis approach.

Specific comments and recommendations follow:

Abstract: the highly detailed abstract conveys much of the essential information about the study, allowing the reader to gain a clear understanding of the study design, population and context, methods and major findings. The one important detail missing is the age group of the study population; I recommend adding the full age range to provide that one important detail. A mean age of 22.5 is referenced for the ‘fellows’; this suggests a primarily young adult population (eg., early to mid 20s) rather than a specifically adolescent population. This is likely because the ‘fellows’ are older adolescents (have they graduated from this or another similar program?). If the full age range is included when referring to the adolescent fellows, it will then be clear if the authors are referencing a population that is 14-19, versus 15-24, versus 10-19 (all of which fit within a definition of ‘adolescent’). Also, a brief mention of the age group of the population that is served by the program would help to clarify the age.

Introduction:

The authors make the point that this study ‘positions ALHIV not merely as disadvantaged recipients of care but as individuals with agency’ who are empowered and who can empower other youth. This is an important foundation for a peer mentorship program.

In support of this, was any other framework or theoretical perspective used to support the study? Since the Social Ecological Model is introduced later, it will be good to add a brief reference to that model here, and then elaborate on it later in the full section devoted to the model. Also consider mentioning the SEM in the abstract.

I’m Possible Intervention Description

This detailed overview of the program includes most relevant details. The distinction between ‘fellows’ and ‘peers’ is slightly murky, as many peer education programs refer to the peer educators as ‘peers’. In the context of this study, this choice of terminology is understandable, but perhaps it could be clarified here and above, in the Abstract, that ‘peers’ are in fact the adolescents in care.

How do the adolescents enroll or access the I’m Possible intervention? Are they assigned to participate through their places of residence, or through the clinics providing HIV care?

Social Ecological Model: overall, this is well explained and connected to the study design and research findings.

CCIs or childcare institutions are mentioned as residential spaces for ALHIV – is this typical in India, for ALHIV to be in some type of care facility (and is this due to parental loss from HIV?). Explaining this for readers not familiar with the context would be helpful (and what % of study participants live in such settings?).

Methods

Why were only 15 in-depth interviews conducted – how was the sample size of N=15 for the 2 participant groups decided on, and was saturation reach with the interviews in each group (fellows and supervisors/mentors)

Why were focus group discussions selected as the research method for the adolescent peers to discuss the program? In the introduction of the different research methods (Line 161), the adolescent peers are referred to as representing the ‘individual’ level of the socio-ecological model, with a focus on attitudes, knowledge and skills. Typically, assessment of this type of knowledge would then occur with in-depth interviews, which are better suited to exploration of individual level experiences and attributes. With this younger age group of adolescents, it is understandable that a group setting may have been more feasible and comfortable, but this should be discussed and explained. There should be separate discussions of the FGDs and the IDIs, as the data must be analyzed separately, with attention to group-level dynamics and analysis for the FGDs.

For instance, in the thematic analysis, discussed in lines 220-235, what was learned beyond the level of individual experience? Examples are provided in the research findings, including the essential attributes of peer support (informational, emotional and affirmative).

Research Findings

The main themes of mentorship, peer influence, personal growth, systemic challenges: stigma and mental health, and sustainability through post-fellowship support appear to capture the essence of the APHIV’s general perspectives. Within each theme, the quotations are very short, and there is little comparison of the perspectives of different participants.

Tables 1 and 2 have a number of quotations that could enhance the presentation of findings; I suggest working to develop a richer narrative in this section by using longer quotes, and by adding quotes from Tables 1 and 2 into that section (summarizing the key points from each table and integrating them into themes throughout this section). This would create a more balanced and in-depth presentation of the thematic findings. The tables would be suitable as an appendix to the paper.

There is an opportunity to compare the different types of participants, as well as the different methods (interviews vs focus groups), and to add a paragraph at the end of the research findings to discuss these comparisons (compare and contrast).

Overall, a stronger narrative is needed to address the critiques above, and to strengthen the presentation of research findings.

The Discussion reads well overall, and includes key points from the study. Once the research findings and data analytical approach are deepened and refined, the discussion could be revisited for some additional points to be added, for a more comprehensive discussion of the findings and their implications.
