# Supplementary material for: “I can be a source of motivation”: Perspectives from stakeholders of the I’mPossible fellowship, a peer-led differentiated service delivery model for adolescents with perinatally acquired HIV in India
Source: PLOS Glob Public Health. 2025 Sep 4;5(9):e0004453. doi: 10.1371/journal.pgph.0004453 (PMC12410805; doi:10.1371/journal.pgph.0004453)
Supplement: Attachment — Submitted filename: Response to Comments_June012025.docx [file pgph.0004453.s005.docx]

**Response to Comments (Journal & Reviewer)**

Journal Requirements:

**1. Please include a complete copy of PLOS’ questionnaire on inclusivity in global research in your revised manuscript. Our policy for research in this area aims to improve transparency in the reporting of research performed outside of researchers’ own country or community. The policy applies to researchers who have travelled to a different country to conduct research, research with Indigenous populations or their lands, and research on cultural artefacts. The questionnaire can also be requested at the journal’s discretion for any other submissions, even if these conditions are not met. Please find more information on the policy and a link to download a blank copy of the questionnaire here: https://journals.plos.org/globalpublichealth/s/best-practices-in-research-reporting. Please upload a completed version of your questionnaire as Supporting Information when you resubmit your manuscript.**

Response: The PLOS’ questionnaire on inclusivity in global research has been completed and included in the revised manuscript.

**2. In the online submission form, you indicated that The data analyzed in this study were collected as part of a qualitative research project. Relevant excerpts from participant transcripts are included within the paper. De-identified data used in this study may be accessed upon reasonable request to the corresponding author.**

**All PLOS journals now require all data underlying the findings described in their manuscript to be freely available to other researchers, either**

**1. In a public repository,**

**2. Within the manuscript itself, or**

**3. Uploaded as supplementary information.**

**This policy applies to all data except where public deposition would breach compliance with the protocol approved by your research ethics board. If your data cannot be made publicly available for ethical or legal reasons (e.g., public availability would compromise patient privacy), please explain your reasons by return email and your exemption request will be escalated to the editor for approval. Your exemption request will be handled independently and will not hold up the peer review process, but will need to be resolved should your manuscript be accepted for publication. One of the Editorial team will then be in touch if there are any issues.**

Response:

This study involved qualitative interviews and focus group discussions with youth living with HIV and program stakeholders in India. While anonymized excerpts from transcripts have been included within the manuscript to illustrate key findings, we did not obtain ethical approval to share full transcripts publicly. Participants were informed during the consent process that transcripts would be stored securely and destroyed following the completion and dissemination of the study.

The Johns Hopkins Bloomberg School of Public Health IRB and the YR Gaitonde Centre for AIDS Research and Education (YRG CARE) IRB approved the study with these conditions. Public sharing of full transcripts would violate these approved protocols and pose significant ethical risks, potentially compromising participant confidentiality and safety.

However, we have now included the code book from this analysis as supplementary material. This document lists all the codes that our data analysts used and their code definitions where they were applied. Researchers with specific questions or requests related to the raw data or data analysis may contact the corresponding author at ashet1@jhu.edu, or reach out to the IRBs at jhsph.irboffice@jhu.edu or ethics@yrgcare.org.

**3. Please provide separate figure files in .tif or .eps format.**

**For more information about figure files please see our guidelines:**

**https://journals.plos.org/globalpublichealth/s/figures**

**https://journals.plos.org/globalpublichealth/s/figures#loc-file-requirements**

Response: Figures have been uploaded in .tif format.

**4. Please review your reference list to ensure that it is complete and correct. If you have cited papers that have been retracted, please include the rationale for doing so in the manuscript text, or remove these references and replace them with relevant current references. Any changes to the reference list should be mentioned in the rebuttal letter that accompanies your revised manuscript. If you need to cite a retracted article, indicate the article’s retracted status in the References list and also include a citation and full reference for the retraction notice.**

Response: All references have been checked for completeness and accuracy. Four additional references have been included to ensure that reviewers’ comments were addressed to explain residential status of youth living with HIV in India and the strength of intra-paradigm research. Another reference was added to clarify three elements of peer support.

Additional Editor Comments:

Abstract

**Recommendation: “…the age group of the study population; I recommend adding the full age range to provide that one important detail.”**

Response: The full age ranges for fellows (18–24 years) and peers (12–18 years) participating in the study have been added to lines 45 and 48 of the abstract. These age ranges have also been included in the Methods section under Data Collection (lines 219 and 236).

**Recommendation: “…a brief mention of the age group of the population that is served by the program would help to clarify the age.”**

Response: The I’mPossible Fellowship program, from its inception from 2021 includes participants with a wide age range as follows; fellows: 18–27 years and peers: 8–26 years. This is described in the Methods section under “I’mPossible fellowship and the social-ecological model of health” on lines 149 and 151.

Introduction

**Recommendation: “In support of this, was any other framework or theoretical perspective used to support the study? Since the Social Ecological Model is introduced later, it will be good to add a brief reference to that model here and then elaborate on it later in the full section devoted to the model. Also consider mentioning the SEM in the abstract.”**

Response: We based our study on the Social Ecological Model (SEM), with the unique angle of involving participants representing the first three layers of the SEM. We have added a reference to the SEM in the abstract and introduction sections, with further elaboration retained in the dedicated section with the subheading, “I’mPossible fellowship and the social-ecological model of health”.

I’m Possible Intervention Description

**Recommendation: “The distinction between ‘fellows’ and ‘peers’ is slightly murky, as many peer education programs refer to the peer educators as ‘peers’. In the context of this study, this choice of terminology is understandable, but perhaps it could be clarified here and above, in the Abstract, that ‘peers’ are in fact the adolescents in care.”**

Response: Clarification that “peers” refers to children, adolescents and young adults with HIV in care and beneficiaries receiving the I’mPossible intervention has been added to the abstract (lines 43-44) and to the Methods section under the program description, “The I’mPossible fellowship and the social-ecological model of health” (lines 148-149).

I’mPossible fellowship and the social-ecological model of health

**Recommendation: “CCIs or childcare institutions are mentioned as residential spaces for ALHIV – is this typical in India, for ALHIV to be in some type of care facility (and is this due to parental loss from HIV?). Explaining this for readers not familiar with the context would be helpful (and what % of study participants live in such settings?).”**

Response:

Childcare institutions (CCIs) are residential facilities that provide care, maintenance, and supervision for minor children, often on a 24/7 basis. In the context of our study setting, CCIs refer to residential facilities caring for children who have lost one or both parents due to HIV. Most children living in the CCIs in our setting are born with HIV themselves.

Among our 18 study participants, 15 (83.3%) resided in CCIs, while the remaining 3 (16.7%) lived in community-based households. India has a large proportion of orphaned children due to HIV; in a six-year retrospective analysis of national-level data from 6 high-HIV-prevalence Indian states, from among 9,051 children with HIV, orphans constituted 55%, with 36% and 19% having lost one or both parents, respectively; 95% lived with parents or relatives, and 4% resided in child-care institutions.

This information has been added to the relevant sections in the manuscript (Introduction, Study Participants, Data Collection) on lines 109-113; 191-193; 241-242.

Methods

**Recommendation: “Why were only 15 in-depth interviews conducted – how was the sample size of N=15 for the 2 participant groups decided on, and was saturation reach with the interviews in each group (fellows and supervisors/mentors).”**

Response:

The purpose of the study was to examine experiences of the first batch of ten fellows initiated within the I’mPossible fellowship and triangulate these findings with experiences of peer beneficiaries and institutional supervisors who mentored the fellows. Since the first batch of fellows included 9 who had fully completed the program and 7 supervisors who mentored fellows, we were limited by an inherently small sample size and included only 15 participants (one fellow declined consent citing non-availability). It is noteworthy that this sample represents near-complete coverage of the participant population, and thematic saturation was achieved within both groups of fellows and supervisors. These points are acknowledged in the data Collection and Discussion sections in the manuscript (lines 226-232; 546-549).

**Recommendation: “Why were focus group discussions selected as the research method for the adolescent peers to discuss the program? In the introduction of the different research methods (Line 161), the adolescent peers are referred to as representing the ‘individual’ level of the socio-ecological model, with a focus on attitudes, knowledge and skills. Typically, assessment of this type of knowledge would then occur with in-depth interviews, which are better suited to exploration of individual level experiences and attributes. With this younger age group of adolescents, it is understandable that a group setting may have been more feasible and comfortable, but this should be discussed and explained. There should be separate discussions of the FGDs and the IDIs, as the data must be analyzed separately, with attention to group-level dynamics and analysis for the FGDs.”**

Response: The goal of the FGDs with peers was to (1) encourage participation through a group format that would make peers feel more comfortable speaking, and (2) explore the collective experience of being a peer within the program. While IDIs are often used to explore individual-level attitudes, knowledge, and skills, FGDs were chosen as an exploratory tool for peers to better capture the collective experience of being a peer in this program, as well as to encourage open discussion and peer interaction. To make this distinction clear, we have separated the IDI and FGD descriptions and have added corresponding distinctions in the Data Analysis section to indicate that IDIs with fellows and supervisors were analyzed to explore individual-level experiences and reflections, while FGDs with peers were analyzed separately, with attention to group-level dynamics, shared experiences, and interactions between participants (lines 260-263).

Research Findings:

**Recommendation: “Within each theme, the quotations are very short, and there is little comparison of the perspectives of different participants.**

**Tables 1 and 2 have a number of quotations that could enhance the presentation of findings; I suggest working to develop a richer narrative in this section by using longer quotes, and by adding quotes from Tables 1 and 2 into that section (summarizing the key points from each table and integrating them into themes throughout this section). This would create a more balanced and in-depth presentation of the thematic findings. The tables would be suitable as an appendix to the paper.**

**There is an opportunity to compare the different types of participants, as well as the different methods (interviews vs focus groups), and to add a paragraph at the end of the research findings to discuss these comparisons (compare and contrast).”**

Response: As per the reviewer’s comments, we have added additional quotations and expanded the text to present more narrative, in-depth views. We have integrated selected quotes from the tables into the Results section of the thematic analysis. We also highlight the triangulation approach of analyzing perspectives from three different types of stakeholders and note that we did not observe fundamentally differing perspectives across participant groups. Rather, each group contributed complementary insights that collectively deepened the understanding of the program’s impact (lines 446-450).

Additionally, in response to these comments, we added a new ‘compare and contrast’ paragraph to the Discussion section highlighting the strength of incorporating multi-perspective approaches and multiple qualitative methods in evaluating the intervention (lines 526-542).
